# First report in South America of companion animal colonization by the USA1100 clone of community-acquired meticillin-resistant *Staphylococcus aureus* (ST30) and by the European clone of methicillin-resistant *Staphylococcus pseudintermedius* (ST71)

**DOI:** 10.1186/1756-0500-6-336

**Published:** 2013-08-27

**Authors:** Isidório Mebinda Zuco Quitoco, Mariana Severo Ramundo, Maria Cícera Silva-Carvalho, Raquel Rodrigues Souza, Cristiana Ossaille Beltrame, Táya Figueiredo de Oliveira, Rodrigo Araújo, Pedro Fernandez Del Peloso, Leonardo Rocchetto Coelho, Agnes Marie Sá Figueiredo

**Affiliations:** 1 Departamento de Microbiologia Médica, Universidade Federal do Rio de Janeiro, Instituto de Microbiologia Paulo de Góes, Av. Carlos Chagas Filho 373, Centro de Ciências da Saúde - Bloco I, Rio de Janeiro 21941-912, RJ, Brazil; 2 Universidade Federal Fluminense, Faculdade de Veterinária, Av. Ary Parreiras 64, Niterói 24230-340, RJ, Brazil; 3 Espaço Pet Veterinária, Av. das Américas 6700, Rio de Janeiro 22793-081, Brazil; 4 Laboratório Richet, Av. das Américas 4801, Rio de Janeiro 22271-110, RJ, Brazil; 5 Current address: Universidade Federal do Rio de Janeiro, Campus UFRJ-Macaé Prof. Aloísio Teixeira, Rua Aluísio da Silva Gomes, 50, Granja dos Cavaleiros, Polo Universitário - Macaé, Rio de Janeiro 27930-560, RJ, Brazil

**Keywords:** CA-MRSA, Companion animals, *S. aureus*, Clonality, MRCoNS, *S. pseudintermedius*, *S. saprophyticus*

## Abstract

**Background:**

Methicillin-resistant staphylococci can colonize and cause diseases in companion animals. Unfortunately, few molecular studies have been carried out in Brazil and other countries with the aim of characterizing these isolates. Consequently, little is known about the potential role of companion animals in transmitting these resistant bacteria to humans. In this work we searched for *mecA* gene among *Staphylococcus* isolates obtained from nasal microbiota of 130 healthy dogs and cats attended in a veterinary clinic located in the west region of Rio de Janeiro. The isolates recovered were identified to the species level and characterized using molecular tools.

**Results:**

A community-acquired methicillin-resistant *Staphylococcus aureus* (CA-MRSA) isolate related to USA1100 (Southwest Pacific clone) and susceptible to all non-β-lactams was detected in a cat (1.7%, 1/60). Another coagulase-positive isolate harboring *mecA* was recovered from a dog (1.4%, 1/70) and identified as *Staphylococcus pseudintermedius* (MRSP) related to the European clone (ST71). The two isolates of *Staphylococcus conhii* subsp. *urealyticus* (1.4%, 1/70 dogs and 1.7%, 1/60 cats), similarly to the MRSP isolate, also presented high-level multiresistance. The majority of the methicillin-resistant coagulase-negative staphylococci recovered were *Staphylococcus saprophyticus* (5.7%, 4/70 dogs and 6.7%, 4/60 cats) and all clustered into the same PFGE type.

**Conclusions:**

This work demonstrates that *mecA*-harboring *Staphylococcus* isolates are common members of the nasal microbiota of the healthy companion animals studied (9.2%, 12/130 animals), including some high-level multiresistant isolates of *S*. *pseudintermedius* and *S. conhii* subsp. *urealyticus*. The detection, for the first time in South America, of USA1100-related CA-MRSA and of ST71 MRSP (European clone), colonizing companion animals, is of concern. Both *S. pseudintermedius* and *S. aureus* are important agents of infections for animals. The USA1100 CA-MRSA is a causative of severe and disseminated diseases in healthy children and adults. Additionally, MRSP is a nosocomial pathogen in veterinarian settings. It had already been demonstrated that the virulent ST71 MRSP is geographically spread over Europe and USA, with potential for zoonotic infections.

## Background

The end of the 1980 decade was marked by the emergency of community-acquired methicillin-resistant *Staphylococcus aureus* (CA-MRSA) among human populations from a remote area in Australia [1]. The first strain reported was named Western-Australia 1 (WA-1) and later genotyped as ST1-SCC*mec* IV. WA-1 isolates did not frequently carry the genes *lukSF*, encoding for the two subunits (S and F) of the Panton-Valentine leukocidin (PVL) [1,2]. Soon after, CA-MRSA clones spread over different countries [3]. In the USA, three main PVL-positive CA-MRSA clones have been prevailing: USA 300 (ST8-SCC*mec* IV), USA 400 (ST1-SCC*mec* IV) and USA1100 (ST30-SCC*mec* IV), with USA 300 isolates (ST8-SCC*mec* IV, PVL^+^) ranking first among the causatives of skin/soft skin infections in healthy individuals from the community [4-6]. In Brazil, there are few reports on CA-MRSA infections and most of the isolates are related to USA 1100, which have been detected causing infections ranging from simple and localized to severe and invasive diseases, in previously healthy children and adults [7]. More recently, MRSA isolates have been found colonizing and infecting both companion and livestock animals with clear economical and public health implications [8-10]. The detection of MRSA in animals has raised concerns among scientists over their role as potential reservoirs or vectors for CA-MRSA infections in humans. Besides MRSA, other methicillin-resistant staphylococci (MRS) have recently been detected among companion animals such as methicillin-resistant *Staphylococcus pseudintermedius* (MRSP). *S. pseudintermedius* is a major cause of purulent and opportunistic infections in dogs, such as dermatitis, otitis and wound infections. Indeed, MRSP are frequently multidrug resistant pathogens resembling typical hospital isolates [11,12].

Methicillin-resistant coagulase negative staphylococci (MRCoNS) have also been isolated in low frequencies from small companion animals and horses [13,14]. *Staphylococcus saprophyticus*, for example, is a common agent of uncomplicated urinary tract infection (UTI) in young women [15]. Although the report of *mecA*-harboring *S. saprophyticus* is very rare, these isolates have recently been detected from human patients presented with UTI [16,17]. Besides, MRCoNS are recognized as opportunistic pathogens for immunocompromized patients, including high-risk human neonates [18]. Of concern is the fact that MRCoNS are considered a potential reservoir of resistance determinants, including staphylococcal cassette *mec*[17]. In the study presented here we searched for *mecA*-harboring *Staphylococcus* isolates in the nasal microbiota of 130 companion animals attended in a veterinary clinic in Rio de Janeiro. The isolates recovered were identified at subspecies level, and the antimicrobial susceptibility and genotypic profiles determined.

## Results and discussion

The *mecA* gene was confirmed in the 12 staphylococci that grew in the enrichment broth (9.2%, 12/130 animals). From these methicillin-resistant streptococci (MRS) isolates 10 were MRCoNS (7.7%, 10/130) and 2 coagulase-positive MRS (1.5%, 2/130). The majority of MRCoNS were identified as *Staphylococcus saprophyticus* (80%, 8/10) and the remaining as *Staphylococcus conhii* ssp. *urealyticus* (20%, 2/10). One coagulase-positive isolate (BMBSA87), collected from a cat, harbored *mecA* and was identified as MRSA (8.3%, 1/12) and the other isolate (BMBSP02), obtained from a dog, was initially identified as *Staphylococcus intermedius* by the automated method (8.3%, 1/12). Because all dog isolates identified thus far by routine phenotypic tests as *S. intermedius* clustered into the newly identified species *S. pseudintermedius*[19], additional PCR identification was carried out yielding an expected single band of 926 bp, confirming the classification of this isolate as *S. pseudintermedius*.

The MRSA isolate was susceptible to all drugs tested except β-lactams. All isolates identified as *S. saprophyticus* (except one that was also resistant to rifampicin; 7/8; 87.5%) displayed antimicrobial resistance only to erythromycin and clindamycin, in addition to β-lactams. Despite that, the MRSP isolate was resistant to ciprofloxacin, clindamycin, erythromycin, gentamicin and trimethoprim sulphamethoxazole, in addition to β-lactams. Multiresistance was also detected in 2 isolates of *S*. *conhii* ssp. *urealyticus* (Table 1).

**Table 1:** **Species identification and antimicrobial susceptibility profiles of methicillin-resistant *Staphylococcus *isolated from healthy companion animals**

**Isolate**	**Species**	**Animal**	**Antimicrobial susceptibility**^**a**^											
			**CEF**	**CIP**	**CLI**	**CLH**	**ERY**	**GEN**	**OXA**	**PEN**	**RIF**	**TEI/ VAN**	**TET**	**TMP**
BMBSP02	*S. pseudintermedius*	Dog	R^b^	R	R	R	R	R	R	R	S	S	S	R
BMBSA87	*S. aureus*^c^	Cat	R	S	S	S	S	S	R	S	S	S	S	S
BMBSC28	*S. conhii*-urea^d^	Cat	R	S	R	S	R	R	R	R	R	S	S	R
BMBSC30	*S. conhii*-urea	Dog	R	R	R	R	R	R	R	R	S	S	S	S
BMBSS05	*S. saprophyticus*	Dog	R	S	R	S	R	S	R	R	S	S	S	S
BMBSS18	*S. saprophyticus*	Cat	R	S	R	S	R	S	R	R	R	S	S	S
BMBSS21	*S. saprophyticus*	Dog	R	S	R	S	R	S	R	R	S	S	S	S
BMBSS35	*S. saprophyticus*	Cat	R	S	R	S	R	S	R	R	S	S	S	S
BMBSS106	*S. saprophyticus*	Cat	R	S	R	S	R	S	R	R	S	S	S	S
BMBSS110	*S. saprophyticus*	Cat	R	S	R	S	R	S	R	R	S	S	S	S
BMBSS116	*S. saprophyticus*	Dog	R	S	R	S	R	S	R	R	S	S	S	S
BMBSS130	*S. saprophyticus*	Dog	R	S	R	S	R	S	R	R	S	S	S	S

^a^Antibiograms were carried out as described by the CLSI for the following antimicrobial drugs: cefoxitin (CEF), ciprofloxin (CIP), clindamycin (CLI), chloramphenicol (CHL), erythromycin (ERY), gentamicin (GEN), penicillin G (PEN), rifampicin (RIF), sulfamethoxazole-trimethoprim (TMP), teicoplanin (TEC) and tetracycline (TET). All isolates were susceptible to vancomycin (VAN) when MIC was determined.

^b^*R*: resistant and *S*: susceptible.

^c^*S.aureus*: *Staphylococcus aureus* ssp. *aureus*.

^d^*S. conhii*-urea: *S. conhii* ssp. *urealyticus.*

**Figure 1:**
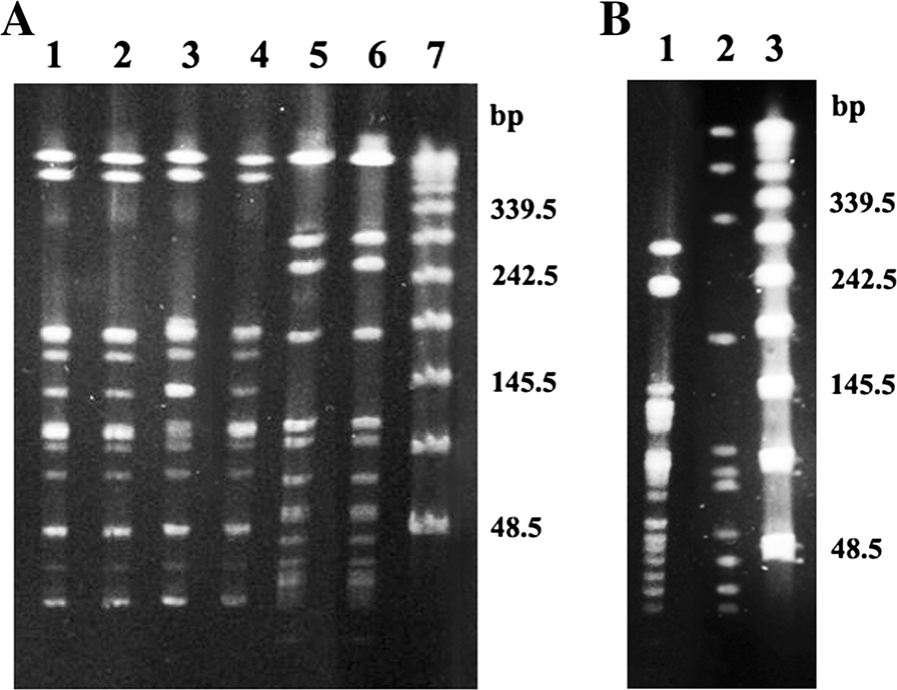
**Pulsed-field gel electrophoresis (PFGE) of the *Sma*I-fragmented genomic DNA of the methicillin-resistant *Staphylococcus *isolates obtained from nasal swabs of companion animals. A**, lanes 1–4: Representives of the methicillin-resistant *S. saprophyticus* ; 5: MRSA from cat (isolate 87); 6: Human isolate of CA-MRSA belonging to USA 1100 (ST30-SCC*mec* IV); 7: Lambda-ladder molecular size marker. **B**, lane 1: *S. pseudintermedius* from dog (isolate 02); 2: Representative isolate of the *S. conhii*-urea (isolate 28) and 3: Lambda-ladder molecular size marker.

The prevalence of MRS among dogs was as follows: 1.4% (1/70) carried MRSP, 1.4% (1/70) methicillin-resistant *S. conhii*-urea (MRSC) and 5.7% (4/70) methicillin-resistant *S. saprophyticus* (MRSS), and among cats was: 1.7% (1/60) carried MRSA, 1.7% (1/60) MRSC and 6.7% (4/60) MRSS. The MRSA isolate BMBSA87 was typed as SCC*mec* IV and displayed a PFGE pattern indistinguishable to that of CA-MRSA isolate WB49, a representative of the USA1100 clone (ST30-SCC*mec* IV; Figure 1A, Lanes 5–6), previously detected in Porto Alegre, RS, Brazil, from human skin/soft skin infection [20]. In addition, isolate BMBSA87 displayed the multilocus sequencing type (MLST) allelic profile: arcc = 2, aroe = 2, glpf = 2, gmk_ = 2, pta_ = 6, tpi_ = 3, yqil = 2, corresponding to ST30. However, amplification for the genes *lukSF* (encoding for PVL) could not be detected when total DNA of this isolate was tested with two different, specific, set of primers. PVL is commonly produced by ST30-SCC*mec* IV isolates [21,22]. Despite that, cases of skin/soft tissue infections (SSTI) associated with PVL-negative CA-MRSA isolates have been reported [22,23]. In addition, the MRSA isolate detected in the present study harbored *pmsα3* encoding for the phenol soluble moduline α3, which has been associated with the pathogenesis of SSTI [24]. Our data showed that this MRSA isolate also carried the enterotoxin genes *seg*, *sei*, *seo* of the *egc* cluster, a highly prevalent operon of enterotoxin genes. It was suggested that the apparent redundancy of these superantigens provides selective advantage towards bacterial colonization and/or spread of the host and not only for toxemia [25]. The rate of MRSA colonization in dogs (1.4%, 1/70) was similar to those obtained in other studies (about 1- 2%) carried out in Hong Kong (2.1%, 15/704) and UK (0.7%, 6/850) [26,27]. Indeed, small number of MRSA was detected as cause of urinary tract infections in dogs in Brazil (0.9%, 3/348) [13]. This incidence is in accordance with previous data reporting low percentage (1.2%) of CA-MRSA carriers among human infant in Brazil [28].

*S. pseudintermedius* is an important opportunistic pathogen of companion animals. It is the most frequent *Staphylococcus* isolates collected from infected dogs. Since 2006 there has been a significant emergence of MRSP [12]. Many of MRSP isolates from Europe and USA (about 54%) were classified as ST71-SCC*mec*II*,* III or NT (NT: nontypable) [11,12]. The MRSP recovered here from dog (isolate BMBSP02) was typed as SCC*me*c NT. Additionally, the lambda-ladder molecular size marker was used as reference to calculate the sizes of the PFGE bands (Figure 1B) of the isolate BMBSP02. These values were compared with those obtained from PFGE images of the MRSP isolates published by Ruscher et al. [12] and Soedarmanto et al. [29], and it was found that the banding patterns were quite similar. Thus, MLST analysis was then carried out and confirmed the presence of this international virulent MRSP clone (European clone) in our country, since the BMBSP02 clustered into ST71, corresponding to the MLST allelic profile ack = 3, cpn60 = 9, fdh = 1, pta = 2, sar = 2, tuf = 1. In Brazil, oxacillin resistance had already been described among *S. pseudintermedius* isolates causing urinary tract infections (8.6%, 6/70 dogs) and external otitis (14.3%, 13/91 dogs), on the basis of oxacillin 1 μg disk. However, these isolates were not fully molecular characterized [13,30]. In a large study, carried out in two distinct localities in South China, with 785 infected or colonized companion animals (612 dogs and 173 cats), it was found a total of 8.8% (69/785) of MRSP, which represented 47.9% (69/144) of the total *S. pseudintermedius* isolates [31]. It is impressive the high level of multiresistance showed by MRSP isolates. In fact, increased antibiotic resistance has recently been a tendency among MRSP isolates of the international clonal complex [12] and cross-transmission dog-owner of a multiresistant MRSP has already been reported [29].

**Figure 2:**
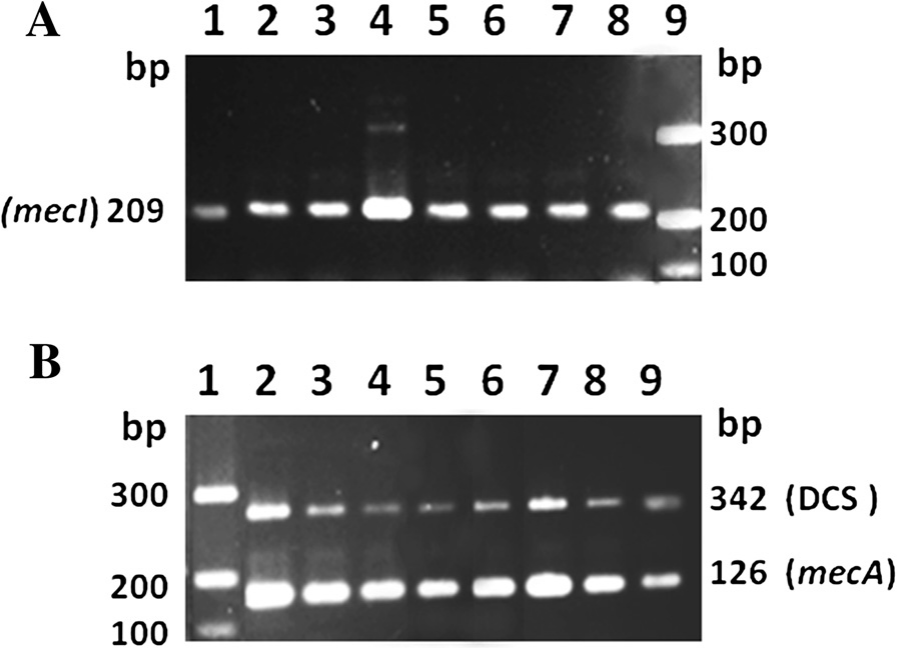
**SCC*mec *typing of *S. saprophyticus *isolates.** Note that all isolates amplified with both *mecI***(A**; Lines 1–8**)** and DCS primers **(B**; Lines 2–9**)**. *mecA* was used as positive control of the PCR reactions. **A**; Line 9 and **B**, Line 1: Low range molecular size marker.

All eight MRSS isolates, from both dogs and cats, clustered into the same PFGE clone type (Figure 1A; lanes 1–4) and generated amplification products for both DCS and *mecI* primers described in 2007 by Milheiriço et al. [32] (Figure 2). These data indicated that the genes around *mec* complex were similar to those of SCC*mec* II and III (type II-III). Interestingly, eight MRSS isolates detected in Japan, which were nontypeable by the current classification for MRSA or by SCC*mec* sequencing, had the novel *mec* type II-III [16]. Dispite the fact that MRSS isolates were the most frequent MRS colonizing both cats and dogs in our study, *S. saprophyticus* have been isolated in low frequency from infected cats and dog [33]. It is well known that in young women, *S. saprophyticus* is a common agent of urinary tract infections [15], but still its relative importance is low compared with *E. coli*[34]. In a study conducted during one year in Sweden, with women and men of all ages, growth of *S. saprophyticus* was a quite uncommon finding [34]. The majority of individuals with *S. saprophyticus* were women between 15–29 years old (63.8%). However it is important to mention that, in this group, *S. saprophyticus* constituted 12.5% of all urinary tract pathogens [34]. It is also of significance that although still rare, isolates of MRSS have already been reported infecting humans in few countries as Canada [35], USA [36], Japan [16], Sweden [17], and also Brazil [37].

The PFGE type of a representative of *S*. *conhii* ssp. *urealyticus* (*S. conhii*-urea) is presented in Figure 1B (lane 2). Similarly to the MRSP isolate, *S. conhii*-urea isolates could not be typed for SCC*mec* with the type system used. Although very rare and generally considered a commensal bacteria, the presence of *mecA* has already been described in *S. conhii* either from human or animal origins [38].

## Conclusions

This work demonstrates that *mecA*-harboring *Staphylococcus* isolates are common members of the nasal microbiota of the companion animals examined. Some bacterial species recovered are involved not only in animals but also in human infections. Moreover, the presence of USA1100-related MRSA colonizing animals is of concern. ST30 isolates are important CA-MRSA pathogens worldwide spread that has been associated to serious invasive diseases in adult and children in this country [39]. *S. pseudintermedius* is the most prevalent pathogenic streptococci in companion animals. To the best of our knowledge, that is the first report recognizing the prevalence of the European clone of MRSP (ST71) and of the USA1100 clone of MRSA (ST30) colonizing animals in Brazil. Both CA-MRSA and MRSP have already been incriminated in potential zoonotic diseases, reflecting the recent emergency of these important multidrug-resistant pathogens in animals.

The fact that most of *mecA*-positive isolates were *S. saprophyticus* also poses an important question concerning potential transfer of MRSS between animals and humans. Finally, our data indicate that continued surveillance studies involving fully identification and genotyping of *Staphylococcus* isolates should be carried out in this and other countries for monitoring the spread of MRS among companion animals.

## Methods

### Sample collection

Hundred and thirty nasal swabs were obtained from 70 dogs and 60 cats which were visiting a veterinarian clinic, located in Rio de Janeiro city, RJ, for pet grooming, during the year of 2010. The animals included in this study were healthy and free of hospitalization, antimicrobial therapy and invasive device in the six-month before sample collections. The swabs were collected from the anterior nares of the animals, using a transport system (Culture Swab Transport System; Copan Italia SpA.; Brescia Italy), and stored at 4°C for no more than 24 h. Written informed consent for participation was obtained from the owners of all animals included in the study. The study was approved (# IMPPG030) by the Animal Ethics Committee from Centro de Ciências da Saúde, Universidade Federal do Rio de Janeiro.

### Bacterial identification

In the laboratory, the swab was introduced in an enrichment broth constituted of tryptic soy broth (TSB, BD; Becton, Dickinson and Company, BD; Becton DriveFranklin Lakes, NJ, USA) supplemented with 10 μg/mL methicillin and 7.5% of NaCl [40]. After incubation at 37°C for 18 h-48 h, aliquots of the turbid cultures were streaked on mannitol salt agar (BD; Becton, Dickinson and Company, BD; Becton Drive Franklin Lakes, NJ, USA) and incubated at 37°C for 18 h-24 h. Tube coagulase test was carried out for Gram-positive and catalase-positive colonies. Both coagulase-positive and -negative staphylococci were identified using Microscan WalkAway 96 SI automated system (Siemens Healthcare Diagnostic, West Sacramento, CA, USA) using Pos Combo Panel Type 33 (PC33) for bacterial identification and susceptibility tests. PCR amplification using the set of primers pse-F2 TRGGCAGTAGGATTCGTTAA and pse-R5 CTTTTGTGCTYCMTTTTGG was carried out for molecular identification of *S. pseudintermedius*, as previously described [41].

### Antimicrobial susceptibility testing

Antibiograms were also carried out following the Clinical Laboratory Standard Institute (CLSI) [42] recommendations for the following antimicrobial disks: cefoxitin (30 μg; CEF) ciprofloxacin (5 μg; CIP), clindamycin (2 μg; CLI), chloramphenicol (30 μg; CHL), erythromycin (15 μg; ERY), gentamicin (10 μg; GEN), penicillin G (10 UI; PEN), rifampicin (5 μg; RIF), sulfamethoxazole-trimethoprim (25 μg; TMP), teicoplanin (30 μg; TEC) and tetracycline (30 μg; TET). The minimum inhibitory concentration (MIC) was determined for vancomycin (Sigma-Aldrich, St. Louis, MO, USA) according to CLSI guidelines [42]. The *mecA* was tested by polymerase chain reaction (PCR)-amplification of an internal fragment of the gene [43]. *Staphylococcus aureus* ATCC 25923 and *S. aureus* ATCC 29213 were used for quality control purposes.

### Bacterial genotyping

SCC*mec* typing was carried out for all isolates using different typing systems [32,43-46]. Pulsed-field gel electrophoresis (PFGE) of the *Sma*I-fragmented DNA was carried out for all isolates recovered, according to Teixeira et al. [47]. Multilocus sequence typing (MLST) was carried out as recommended for MRSA [48] and *S. pseudintermedius*[49] isolates. To assign the sequence types the allele sequences were correctly trimmed and submitted to the *S. aureus* MLST database (http://www.mlst.net), for the CA-MRSA BMBSA87, or to the MLST website (http://pubmlst.org/spseudintermedius/) sited at the University of Oxford for *S. pseudintermedius*[50].

### Detection of virulence genes

The gene *lukF-PV* and those encoding for enterotoxins G, I, N and O of the *egc* cluster (*seg*, *sei*, *sen* and *seo*) were assessed by PCR, as described previously [20]. Additionally, another set of primers was used to test the genes *lukSF-PV*[21]. The *pmsα*3 was tested with the primers described by Li et al. [25].

## Abbreviations

CA-MRSA: Community-acquired methicillin-resistant *Staphylococcus aureus*; CEF: Cefoxitin; CIP: Ciprofloxacin; CHL: Chloramphenicol; CLI: Clindamycin; CLSI: Clinical Laboratory Standard Institute; CoNS: Coagulase-negative staphylococci; egc: Enterotoxin gene cluster; ERY: Erythromycin; GEN: Gentamicin; MLST: Multilocus locus sequence typing; MRS: Methicillin-resistant staphylococci (MRS); MRSA: Methicillin-resistant *Staphylococcus aureus*; MRCoNS: Methicillin-resistant coagulase negative staphylococci; MRSC: Methicillin-resistant *Staphylococcus conhii* ssp. *urealyticus*; MRSP: Methicillin-resistant *Staphylococcus pseudintermedius*; MRSS: Methicillin-resistant *Staphylococcus saprophyticus*; PCR: Polymerase chain reaction; PEN: Penicillin G; PFGE: Pulsed-field gel electrophoresis; PVL: Panton-Valentine leukocidin; RIF: Rifampicin; SCCmec: Staphylococcal cassette chromosome *mec*; ST: Sequence Type; SSTI: Skin/soft tissue infections; TEC: Teicoplanin; TET: Tetracycline 30 μg; TMP: Sulfamethoxazole-trimethoprim; TSB: tryptic soy broth; UTI: Urinary tract infections; WA-1: Western-Austrália 1.

## Competing interests

All authors declare that they have no competing interest.

## Authors’ contributions

IMZQ, MSR, MCSC and LCR carried out the bacterial isolation, identification and participate in the majority of the experiments of this paper. COB and RRS carried out PFGE analysis. TFO has been involved in the study design and in the draft of the manuscript. RO was responsible for examining and selecting healthy animals, for collecting bacterial specimens and also for revising critically the manuscript with important intellectual contributions. PDP carried out experiments for identification and speciation of the coagulase-negative isolates. AMSF has been involved in the study design and in the revision of the manuscript critically for final approval of this version. All authors read and approved the final manuscript.

## References

[B1] Pearman JW Christiansen KJ Annear DI Goodwin CS Metcalf C Donovan FP Macey KL Bassett LD Powell IM Green JM Harper WE Mckelvie MS Control of methicillin-resistant *Staphylococcus aureus* (MRSA) in an Australian metropolitan teaching hospital complex Med J Aust 1985 142 103 108 3843849

[B2] Udo EE Pearman JW Grubb WB Genetic analysis of community isolates of methicillin-resistant *S. aureus* in Western Australia J Hosp Infect 1993 25 97 108 10.1016/0195-6701(93)90100-E 7903093

[B3] DeLeo FR Otto M Kreiswirth BN Chambers HF Community-associated meticillin-resistant *Staphylococcus aureus* Lancet 2010 375 1557 1568 10.1016/S0140-6736(09)61999-1 20206987 PMC3511788

[B4] Tenover FC Tickler IA Goering RV Kreiswirth BN Mediavilla JR Persing DH MRSA Consortium Characterization of nasal and blood culture isolates of methicillin-resistant *Staphylococcus aureus* from patients in United States Hospitals Antimicrob Agents Chemother 2012 56 1324 1330 10.1128/AAC.05804-11 22155818 PMC3294931

[B5] Mishaan AM Mason EO Jr Martinez-Aguilar G Hammerman W Propst JJ Lupski JR Stankiewicz P Kaplan SL Hulten K Emergence of a predominant clone of community-acquired *Staphylococcus aureus* among children in Houston, Texas Pediatr Infect Dis J 2005 24 201 296 10.1097/01.inf.0000151107.29132.70 15750454

[B6] David MZ Rudolph KM Hennessy TW Boyle-Vavra S Daum RS Molecular epidemiology of methicillin-resistant *Staphylococcus aureus*, rural southwestern Alaska Emerg Infect Dis 2008 14 1693 1699 10.3201/eid1411.080381 18976551 PMC2630737

[B7] Rozenbaum R Sampaio MG Batista GS Garibald AM Terra GM Souza MJ Vieira EN Silva-Carvalho MC Teixeira LA Figueiredo AM The first report in Brazil of severe infection caused by community-acquired methicillin-resistant *Staphylococcus aureus* (CA-MRSA) Braz J Med Biol Res 2009 42 756 760 10.1590/S0100-879X2009005000007 19578704

[B8] Wan MT Fu SY Lo YP Huang TM Cheng MM Chou CC Heterogeneity and phylogenetic relationships of community-associated methicillin sensitive/resistant *Staphylococcus aureus* isolates in healthy dogs, cats and their owners J Appl Microbiol 2012 112 205 213 10.1111/j.1365-2672.2011.05179.x 22008096

[B9] Lin Y Barker E Kislow J Kaldhone P Stemper ME Pantrangi M Moore FM Hall M Fritsche TR Novicki T Foley SL Shukla SK Evidence of multiple virulence subtypes in nosocomial and community-associated MRSA genotypes in companion animals from the upper midwestern and northeastern United States Clin Med Res 2011 9 7 16 10.3121/cmr.2010.944 20739580 PMC3064756

[B10] Broens EM Espinosa-Gongora C Graat EA Vendrig N Van Der Wolf PJ Guardabassi L Butaye P Nielsen JP De Jong MC Van De Giessen AW Longitudinal study on transmission of MRSA CC398 within pig herds BMC Vet Res 2012 8 58 10.1186/1746-6148-8-58 22607475 PMC3532224

[B11] Perreten V Kadlec K Schwarz S Andersson UG Finn M Greko C Moodley A Kania SA Frank LA Bemis DA Franco A Iurescia M Battisti A Duim B Wagenaar JA van Duijkeren E Weese JS Fitzgerald JR Rossano A Guardabassi L Clonal spread of methicillin-resistant *Staphylococcus pseudintermedius* in Europe and North America: an international *multicentre study* J Antimicrob Chemother 2010 65 1145 1154 10.1093/jac/dkq078 20348087

[B12] Ruscher C Lübke-Becker A Semmler T Wleklinski-Claus G Paasch A Soba A Stamm I Kopp P Wieler LH Walther B Multidrug-resistant *Staphylococcus pseudintermedius* (MRSP) genetic lineage in Europe Vet Microbiol 2010 144 340 346 10.1016/j.vetmic.2010.01.008 20181441

[B13] Penna B Varges R Martins R Martins G Lilenbaum W In vitro antimicrobial resistance of staphylococci isolated from canine urinary tract infection Can Vet J 2010 51 738 742 20885826 PMC2885114

[B14] Bagcigil FA Moodley A Baptiste KE Jensen VF Guardabassi L Occurrence, species distribution, antimicrobial resistance and clonality of methicillin- and erythromycin-resistant staphylococci in the nasal cavity of domestic animals Vet Microbiol 2007 121 307 15 10.1016/j.vetmic.2006.12.007 17270365

[B15] Palou J Pigrau C Molina I Ledesma JM Angulo J Grupo Colaborador Español del Estudio ARESC Etiology and sensitivity of uropathogens identified in uncomplicated lower urinary tract infections in women (ARESC Study): implications on empiric therapy Med Clin 2011 136 1 7 10.1016/j.medcli.2010.02.042 20889171

[B16] Higashide M Kuroda M Omura CT Kumano M Ohkawa S Ichimura S Ohta T Methicillin-resistant *Staphylococcus saprophyticus* isolates carrying staphylococcal cassette chromosome *mec* have emerged in urogenital tract infections Antimicrob Agents Chemother 2008 52 2061 2068 10.1128/AAC.01150-07 18362191 PMC2415764

[B17] Söderquist B Berglund C Methicillin-resistant *Staphylococcus saprophyticus* in Sweden carries various types of staphylococcal cassette chromosome *mec* (SCC*mec*) Clin Microbiol Infect 2009 15 1176 1178 10.1111/j.1469-0691.2009.02771.x 19456833

[B18] Svensson K Hellmark B Söderquist B Characterization of SCC*mec* elements in methicillin-resistant *Staphylococcus epidermidis* isolated from blood cultures from neonates during three decades APMIS 2011 119 885 93 10.1111/j.1600-0463.2011.02801.x 22085365

[B19] Sasaki T Kikuchi K Tanaka Y Takahashi N Kamata S Hiramatsu K Reclassification of phenotypically identified *Staphylococcus intermedius* strains J Clin Microbiol 2007 45 2770 2778 10.1128/JCM.00360-07 17596353 PMC2045239

[B20] Ribeiro A Dias C Silva-Carvalho MC Berquó L Ferreira FA Santos RN Ferreira-Carvalho BT Figueiredo AM First report of infection with community-acquired methicillin-resistant *Staphylococcus aureus* in South America J Clin Microbiol 2005 43 1985 1988 10.1128/JCM.43.4.1985-1988.2005 15815039 PMC1081335

[B21] Jarraud S Mougel C Thioulouse J Lina G Meugnier H Forey F Nesme X Etienne J Vandenesch F Relationships between *Staphylococcus aureus* genetic background, virulence factors, *agr* groups (alleles), and human disease Infect Immun 2002 70 631 641 10.1128/IAI.70.2.631-641.2002 11796592 PMC127674

[B22] Ribeiro A Coronado AZ Silva-Carvalho MC Ferreira-Carvalho BT Dias C Rozenbaum R Del Peloso PF da Costa Ferreira Leite C Teixeira LA Figueiredo AM Detection and characterization of international community-acquired infections by methicillin-resistant *Staphylococcus aureus* clones in Rio de Janeiro and Porto Alegre cities causing both community- and hospital-associated diseases Diagn Microbiol Infect Dis 2007 59 339 345 10.1016/j.diagmicrobio.2007.05.007 17662563

[B23] Srinivasan A Seifried S Zhu L Bitar W Srivastava DK Shenep JL Bankowski MJ Flynn PM Hayden RT Short communication: methicillin-resistant *Staphylococcus aureus* infections in children and young adults infected with HIV AIDS Res Hum Retroviruses 2009 25 1219 1224 10.1089/aid.2009.0040 20001313 PMC2858900

[B24] Li M Cheung GY Hu J Wang D Joo H-S DeLeo FR Otto M Comparative analysis of virulence and toxin expression of global community-associated MRSA strains J Infect Dis 2010 202 1866 1876 10.1086/657419 21050125 PMC3058913

[B25] Jarraud S Peyrat MA Lim A Tristan A Bes M Mougel C Etienne J Vandenesch F Bonneville M Lina G *egc*, a highly prevalent operon of enterotoxin gene, forms a putative nursery of superantigens in *Staphylococcus aureus* J Immunol 2001 166 669 677 11123352 10.4049/jimmunol.166.1.669

[B26] Boost MV Donoghue MMO James A Prevalence of *Staphylococcus aureus* carriage among dogs and their owners Epidemiol Infect 2008 136 953 964 17678561 10.1017/S0950268807009326 PMC2870875

[B27] Loeffler A Pfeiffer DU Lindsay JA Magalhães RJ Lloyd DH Prevalence of and risk factors for MRSA carriage in companion animals: a survey of dogs, cats and horses Epidemiol Infect 2010 14 1 10 10.1017/S095026881000227X 20943000

[B28] Lamaro-Cardoso J de Lencastre H Kipnis A Pimenta FC Oliveira LS Oliveira RM Nouer SS Aires-de-Sousa M Milheiriço C Andrade AL Molecular epidemiology and risk factors for nasal carriage of *staphylococcus aureus* and methicillin-resistant *S. aureus* in infants attending day care centers in Brazil J Clin Microbiol 2009 47 3991 3997 10.1128/JCM.01322-09 19828745 PMC2786686

[B29] Soedarmanto I Kanbar T Ülbegi-Mohyla H Hijazin M Alber J Lämmler C Akineden Ö Weiss R Moritz A Zschöck M Genetic relatedness of methicillin-resistant *Staphylococcus pseudintermedius* (MRSP) isolated from a dog and the dog owner Res Vet Sci 2011 91 25 27 10.1016/j.rvsc.2010.07.025 21353270

[B30] Penna B Varges R Medeiros L Martins GM Martins RR Lilenbaum W Species distribution and antimicrobial susceptibility of staphylococci isolated from canine otitis externa Vet Dermatol 2010 2 292 296 20042036 10.1111/j.1365-3164.2009.00842.x

[B31] Feng Y Tian W Lin D Luo Q Zhou Y Yang T Deng Y Liu YH Liu JH Prevalence and characterization of methicillin-resistant *Staphylococcus pseudintermedius* in pets from South China Vet Microbiol 2012 160 517 524 10.1016/j.vetmic.2012.06.015 22770517

[B32] Milheiriço C Oliveira DC de Lencastre H Update to the multiplex PCR strategy for assignment of *mec* element types in *Staphylococcus aureus* Antimicrob Agents Chemother 2007 51 3374 3377 10.1128/AAC.00275-07 17576837 PMC2043198

[B33] Hauschild T Wójcik A Species distribution and properties of staphylococci from canine dermatitis Res Vet Sci 2007 82 1 6 10.1016/j.rvsc.2006.04.004 17126372

[B34] Eriksson A Giske CG Ternhag A The relative importance of *Staphylococcus saprophyticus* as a urinary tract pathogen: distribution of bacteria among urinary samples analysed during 1 year at a major Swedish laboratory APMIS 2013 121 72 78 10.1111/j.1600-0463.2012.02937.x 23030816

[B35] Hussain Z Stoakes L Massey V Diagre D Fitzgerald V El Sayed S Lannigan R Correlation of oxacillin MIC with *mecA* gene carriage in coagulase-negative staphylococci J Clin Microbiol 2000 38 752 754 10655380 10.1128/jcm.38.2.752-754.2000 PMC86195

[B36] Swenson JM Tenover FC Cefoxitin disk study group. Results of disk diffusion testing with cefoxitin correlate with presence of *mecA* in *Staphylococcus* spp J Clin Microbiol 2005 43 3818 3823 10.1128/JCM.43.8.3818-3823.2005 16081917 PMC1233887

[B37] Souza Antunes AL Secchi C Reiter KC Rodrigues Perez LR Peixoto de Freitas AL Alves d’Azevedo P Evaluation of oxacillin and cefoxitin disks for detection of resistance in coagulase negative staphylococci Mem Inst Oswaldo Cruz 2007 102 719 723 10.1590/S0074-02762007005000078 17924001

[B38] Zong Z Lü X Characterization of a new SCC*mec* element in *Staphylococcus cohnii* PLoS One 2010 5 e14016 10.1371/journal.pone.0014016 21103346 PMC2984492

[B39] de Araújo BE Borchert JM Manhães PG Ferreira FA Ramundo MS Silva-Carvalho MC Seabra AC Victal SH Sá Figueiredo AM A rare case of pyomyositis complicated by compartment syndrome caused by ST30-staphylococcal cassette chromosome *mec* type IV methicillin-resistant *Staphylococcus aureus* Am J Emerg Med 2010 28 537.e3-6 20466258 10.1016/j.ajem.2009.08.010

[B40] Silva FR Mattos EM Coimbra MV Ferreira-Carvalho BT Figueiredo AM Isolation and molecular characterization of methicillin-resistant coagulase-negative staphylococci from nasal flora of healthy humans at three community institutions in Rio de Janeiro City Epidemiol Infect 2001 127 57 62 11561975 10.1017/s095026880100574x PMC2869729

[B41] Sasaki T Tsubakishita S Tanaka Y Sakusabe A Ohtsuka M Hirotaki S Kawakami T Fukata T Hiramatsu K Multiplex-PCR method for species identification of coagulase-positive staphylococci J Clin Microbiol 2010 48 765 769 10.1128/JCM.01232-09 20053855 PMC2832457

[B42] CLSI Performance standards for antimicrobial disk and dilution susceptibility testing; twenty-first informational supplement. M100-S21, Vol. 31 No. 1 2011 PA, USA: Clinical and Laboratory Standards Institute, Wayne

[B43] Oliveira DC de Lencastre H Multiplex PCR strategy for rapid identification of structural types and variants of the *mec* element in methicillin-resistant *Staphylococcus aureus* Antimicrob Agents Chemother 2002 46 2155 2161 10.1128/AAC.46.7.2155-2161.2002 12069968 PMC127318

[B44] Boye K Bartels MD Andersen IS Moller JA Westh H A new multiplex PCR for easy screening of methicillin-resistant *Staphylococcus aureus* SCC*mec* types I-V Clin Microbiol Infect 2007 13 725 727 10.1111/j.1469-0691.2007.01720.x 17403127

[B45] Berglund C Ito T Ikeda M Ma XX Söderquist B Hiramatsu K Novel type of staphylococcal cassette chromosome mec in a methicillin-resistant Staphylococcus aureus strain isolated in Sweden Antimicrob Agents Chemother 2008 52 3512 3516 10.1128/AAC.00087-08 18676883 PMC2565894

[B46] Zhang K McClure JA Elsayed S Conly JM Novel staphylococcal cassette chromosome *mec* type, tentatively designated type VIII, harboring class A *mec* and type 4 *ccr* gene complexes in a Canadian epidemic strain of methicillin-resistant *Staphylococcus aureus* Antimicrob Agents Chemother 2009 53 531 540 10.1128/AAC.01118-08 19064897 PMC2630601

[B47] Teixeira LA Resende CA Ormonde LR Rosenbaum R Figueiredo AM de Lencastre H Tomasz A Geographic spread of epidemic multiresistant *Staphylococcus aureus* clone in Brazil J Clin Microbiol 1995 33 2400 2404 7494036 10.1128/jcm.33.9.2400-2404.1995 PMC228423

[B48] Enright MC Day NPJ Davies CE Peacock SJ Spratt BG Multilocus sequence typing for characterization of methicillin-resistant and methicillin-susceptible clones of *Staphylococcus aureus* J Clin Microbiol 2000 38 1008 1015 10698988 10.1128/jcm.38.3.1008-1015.2000 PMC86325

[B49] Solyman SM Black CC Duim B Perreten V van Duijkeren E Wagenaar JA Eberlein LC Sadeghi LN Videla R Bemis DA Kania SA Multilocus sequence typing for characterization of *Staphylococcus pseudintermedius* J Clin Microbiol 2013 51 306 310 10.1128/JCM.02421-12 23115265 PMC3536184

[B50] Jolley KA Maiden MC BIGSdb Scalable analysis of bacterial genome variation at the population level BMC Bioinformatics 2010 11 595 10.1186/1471-2105-11-595 21143983 PMC3004885

